# The Impact of Pre-Exercise Carbohydrate Meal on the Effects of Yerba Mate Drink on Metabolism, Performance, and Antioxidant Status in Trained Male Cyclists

**DOI:** 10.1186/s40798-022-00482-3

**Published:** 2022-07-16

**Authors:** Thaiana C. Krolikowski, Fernando K. Borszcz, Vilma P. Panza, Laura M. Bevilacqua, Sarah Nichele, Edson L. da Silva, Renata D. M. C. Amboni, Luiz G. A. Guglielmo, Stuart M. Phillips, Ricardo D. de Lucas, Brunna C. B. Boaventura

**Affiliations:** 1 Graduate Program in Nutrition, Health Sciences Center, Federal University of Santa Catarina, Campus Trindade, Florianópolis, SC 88040-370 Brazil; 2 Department of Nutrition, Health Sciences Center, Federal University of Santa Catarina, Campus Trindade, Florianópolis, SC 88040-370 Brazil; 3 Physical Effort Laboratory, Sports Center, Federal University of Santa Catarina, Campus Trindade, Florianópolis, SC 88040-900 Brazil; 4 Department of Clinical Analyses, Health Sciences Center, Federal University of Santa Catarina, Campus Trindade, Florianópolis, SC 88040-370 Brazil; 5 Department of Food Science and Technology, Agricultural Sciences Center, Federal University of Santa Catarina, Campus Itacorubi, Florianópolis, SC 88034-001 Brazil; 6 Department of Kinesiology, McMaster University, Hamilton, ON L8S 4K1 Canada

**Keywords:** Yerba mate, Phenolic compounds, Substrate utilization, Performance, Oxidative stress, Carbohydrate

## Abstract

**Introduction:**

The consumption of yerba mate (YM), a source of antioxidants, in a fasted state increases fatty acid oxidation (FAT_ox_) during low–moderate-intensity exercise and improves performance in high-intensity exercise. However, the impact of a pre-exercise carbohydrate (CHO) meal on YM effects during exercise is unknown.

**Objective:**

We investigated the effects of yerba mate drink (YMD) consumed in the fasted state (YMD-F) or after a CHO meal (YMD-CHO) on measurements of metabolism, performance, and blood oxidative stress markers in cycling exercise.

**Methods:**

In a randomized, repeated-measures, crossover design, eight trained male cyclists ingested (i) YMD-CHO, (ii) YMD-F, or (iii) control-water and CHO meal (Control-CHO). The YMD (an infusion of 5 g of ultrarefined leaves in 250 mL of water) was taken for 7 days and 40 min before exercise. CHO meal (1 g/kg body mass) was consumed 60 min before exercise. The cycling protocol included a 40-min low-intensity (~ 53% *V̇*O_2peak_) constant load test (CLT); a 20-min time trial (TT); and 4 × 10-s *all-out* sprints. Blood samples and respiratory gases were collected before, during, and/or after tests.

**Results:**

During CLT, YMD-CHO increased FAT_ox_ ~ 13% *vs*. YMD-F (*P* = 0.041) and ~ 27% *vs*. Control-CHO (*P* < 0.001). During TT, YMD-CHO increased FAT_ox_ ~ 160% *vs*. YMD-F (*P* < 0.001) and ~ 150% *vs*. Control-CHO (*P* < 0.001). Power output during TT improved ~ 3% (*P* = 0.022) in YMD-CHO *vs*. Control-CHO and was strongly correlated with changes in serum total antioxidant capacity (*r* = −0.87) and oxidative stress index (*r* = 0.76) at post-exercise in YMD-CHO. Performance in sprints was not affected by YMD.

**Conclusion:**

CHO intake did not negate the effect of YMD on FAT_ox_ or TT performance. Instead, a synergism between the two dietary strategies may be present.

*Clinical Trial Registration* NCT04642144. November 18, 2020. Retrospectively registered.

**Supplementary Information:**

The online version contains supplementary material available at 10.1186/s40798-022-00482-3.

## Key Points

- The consumption of yerba mate drink and a pre-exercise carbohydrate meal increased fat oxidation during exercise compared with yerba mate drink intake in the fasted state and control-carbohydrate.
- Yerba mate plus carbohydrate meal increased performance, and this improvement was associated with blood antioxidant status markers. Yerba mate intake in the fasted state did not impair performance in trained male endurance cyclists.
- Yerba mate may be a good dietary strategy for endurance athletes, especially those following methods of periodized carbohydrate intake, which include training sessions with high- and low-carbohydrate availability (i.e., training fasted).

## Introduction

Yerba mate (YM) (*Ilex paraguariensis*) is a polyphenol-rich plant food, which also contains other phytochemicals, including caffeine and saponins [1, 2]. YM tea or drink (YMD) is a very popular beverage consumed in South America and expanded worldwide due to, for example, its sensory characteristics, mainly the bitter taste and aroma, and its contribution to health outcomes [1, 2]. In humans, acute or prolonged YMD consumption has shown antioxidant and anti-inflammatory effects on endogenous and exogenous biomarkers [2–6], hypolipidemic [2, 4, 7, 8] and hypoglycemic [2, 3, 9] actions, and weight loss potential [2, 6, 10, 11]. Moreover, there is evidence that YM can alter substrate metabolism during exercise at low-to-moderate intensities [6, 11–13] and improves time trial cycling performance [12]. The effects of YM on fat oxidation (FAT_ox_) during exercise in humans were first demonstrated by Alkhatib [13].

The potential inhibitory effect of hyperinsulinemia on lipolysis and FAT_ox_ [12, 14, 15] has led to no, or very little, carbohydrate (CHO) being consumed before or during exercise in YM studies [11–13] as the CHO may blunt the pro-lipolytic effects of YM. It is important to emphasize that in previous YM studies [11, 12], the subjects performed exercise tests after fasting overnight (10–12 h). In the overnight fasted state, there is an increase in hepatic glycogenolysis, reduced use of blood glucose by the liver, muscle, and adipose tissue and concomitant hepatic gluconeogenesis. In addition, there is a small but measurable shift toward lipid oxidation [16, 17].

From a practical point of view, exercising after an overnight fast is one of several ways to reduce exogenous CHO availability for training, and it has been incorporated into periodized training nutrition programs (i.e., “train-low” or “train-high”), that aim to promote training-induced adaptations of skeletal muscle (i.e., increased rates of lipid oxidation and improved exercise capacity) [18–20]. Therefore, YM intake after an overnight fast might be a good additional strategy to increment FAT_ox_ and help sustain performance [11–13] in the “train-low” context. However, “train-low” strategies are generally undertaken alongside deliberate training sessions with a high CHO availability period (“train-high”) [18–20]. Hence, the potential ergogenic effects of YM [6, 11–13] should also be addressed in contexts that translate to practical sports strategies with high CHO intake, such as pre-exercise CHO meal [19]. In addition, since YM may improve antioxidant protection [2–5] and strength production involves critical redox-sensitive targets within skeletal muscle [21–23], the effect of YM on the relationships between high-intensity performance and oxidative stress markers may be relevant.

Therefore, we aimed to investigate the effects of the consumption of YMD in a fasted state or after a CHO meal on metabolic and performance variables and oxidative stress biomarkers during an endurance cycling protocol in trained cyclists. We hypothesized that CHO meal would attenuate the effects of YMD on FAT_ox_ during exercise but would improve YMD effects on high-performance cycling.

## Methods

### Subjects and Study Design

This randomized (no placebo) controlled and crossover clinical trial was conducted with an intentional probabilistic sample. Recruited subjects were local cycling and triathlon athletes. The criteria for participation were having at least 2 years of experience with training and competition in cycling endurance events. Individuals with chronic disease, smokers, and those who continuously use medications or dietary supplements who showed aversion, intolerance, or complications resulting from ingesting YMD or any of its components were excluded. Following an explanation of the experimental protocol and its risks, all participants provided written and informed consent. The study was approved by the Human Research Ethics Committee of the Federal University of Santa Catarina (CAAE 04,545,218.1.0000.0121) and was registered at Clinicaltrial.gov (NCT04642144).

**Fig. 1:**
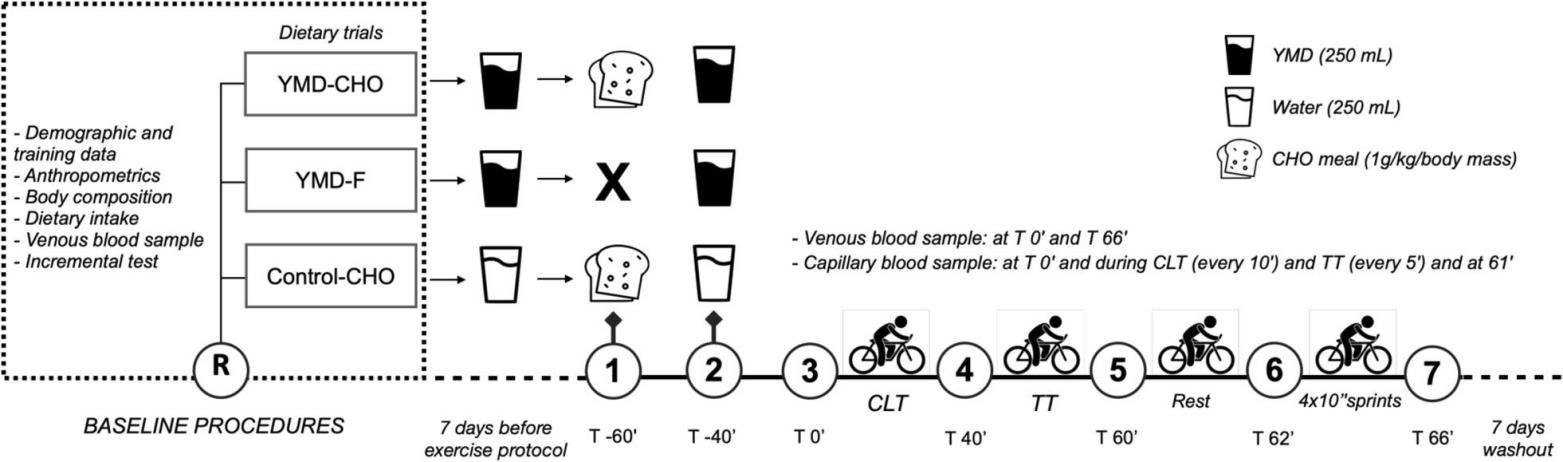
Overview of the study design. The dietary trials order was randomized in a crossover design. R randomization, T time in minutes in relation to the exercise protocol (1–7), *YMD* yerba mate drink, *CHO* carbohydrate, *YMD-F* yerba mate drink and fasting state, *YMD-CHO* yerba mate drink and carbohydrate meal; Control-CHO water and carbohydrate meal, *CLT* constant load test, *TT* time trial

The study comprised four stages: baseline assessment and three trial periods with a 7-day washout between each testing period. The experimental design is shown in Fig. 1. At baseline, subjects reported to the laboratory for demographic and training data collection, anthropometric and body composition assessment, blood sampling, and directions concerning the dietary intake throughout the study. Following this, subjects underwent an incremental cycling test. The order realization of the conditions (i.e., YMD-CHO, YMD-F, and Control-CHO) for each subject was randomized (www.random.org). Participants performed three trials in which the order of realization was randomized: (1) YMD consumption and CHO meal (YMD-CHO) before exercise testing, (2) YMD consumption and fasted state (YMD-F) before exercise testing, and (3) control (without YMD consumption) and CHO meal (Control-CHO) before exercise testing. Participants were instructed to drink 250 mL/day of either YMD or water for straight 7 days (always in the morning), and before the exercise protocol of each of the test periods. On the morning of the following day (8th day), all subjects reported to the laboratory at ~ 8:00 a.m. after a 10-h overnight fast. Exactly 60 min before exercise, during the YMD-CHO and Control-CHO trials, subjects consumed a CHO meal (1 g per kg of body mass) composed of white bread and corn syrup [19], whereas during the YMD-F trial, the subjects remained in a fasted state. At 40 min before exercise, subjects drank 250 mL of either YMD or water according to their trial condition. After this, all subjects performed a ~ 65-min cycling exercise protocol, comprised of a sequence of a constant load test (CLT); a 20-min TT; and a repeated sprint test. Blood samples were obtained at the baseline, before, during, and immediately after the exercise protocol. The experimental protocol was carried out in the same period of the day (± 1 h) to minimize circadian variation effects.

### Body Composition and Health Assessment

Body composition was measured using computed densitometry by dual-energy radiological absorptiometry (Lunar Prodigy Advance General Electric-GE®, Diegem, Belgium). To characterize the health status of the subjects at baseline, routine biochemical parameters (i.e., total cholesterol, triglycerides, high-density lipoprotein cholesterol, low-density lipoprotein cholesterol, urea, creatinine, glucose, uric acid, complete blood count, reactive protein C, alanine aminotransferase, and aspartate aminotransferase) were determined using the Wiener Analyzer (Wiener Laboratórios SAIC, Rosario, Argentina) and Sysmex XE-2100D (Kobe, Japan), according to the each manufacturer’s instruction. Subjects’ values of all variables analyzed were normal (data not presented).

### Yerba Mate Drink Preparation and Ingestion

The dry and ultrarefined green leaves of YM (*Ilex paraguariensis* St. Hil.) were supplied by Indústria Mate Laranjeiras (Laranjeiras do Sul, PR, Brazil). The YMD was prepared with 5 g of leaves with 250 mL of filtered boiling water immediately before intake to minimize the oxidation of the bioactive compounds. The water boiled (~ 90 °C) was immediately poured into the leaves, let rest for 10 min, and then filtered. No sugar, sweetener, or fruits were added to the beverage. For consumption during the 7 days before the exercise protocol test, it was provided for the subject’s packages of yerba mate samples with material and instructions for preparation. YMD was ingested in YMD-F and YMD-CHO trials, both in the morning and daily 7 days before the exercise protocol test and 40 min before the exercise protocol test (Fig. 1).

### Determination of Total Phenolics and Caffeine of Yerba Mate Drink

The total phenolic content was determined spectrophotometrically according to the Folin–Ciocalteu method [24]. Isolated phenolic compounds and caffeine were assessed through high-performance liquid chromatography [25]. The phenolic and caffeine content of 250 mL dose of YMD were: 225.0 ± 12.8 mg of total phenolic content; 12.5 ± 0.9 mg of caffeic acid; 20.0 ± 1.5 mg of *p*-coumaric acid; 37.5 ± 3.1 mg of chlorogenic acid (5-caffeoylquinic acid); and 20.0 ± 1.1 mg of caffeine.

### Dietary and Exercise Control

All subjects were instructed to maintain their habitual dietary intake, throughout the study, except for the suppression of herbal teas/infusion, “*chimarrão/tereré*,” mate tea, or any drink and/or food containing YM. They were also asked to refrain from consuming alcohol and beverages with caffeine (max 200 mg/day). These dietary restrictions were initiated 7 days before baseline assessments and were maintained until the end of all three trials. The usual food consumption of the subjects during the study was assessed through 3-day dietary records (one day weekend and two non-consecutive days during the week), which were completed in each of three trial periods. A registered dietitian provided instructions regarding how to complete the record. Assessment of the subjects’ 3-day dietary records showed no significant differences between the three trial periods for the energy and the analyzed nutrient intakes (Table 1). Subjects were instructed to maintain their usual training routines but refrain from strenuous exercise for 48 h before each exercise protocol.

**Table 1:** Dietary intake from 3-day dietary records at each pre-condition periods

Variable	YMD-CHO	YMD-F	Control-CHO
Energy (kcal)	2,360.7 ± 553.2	2,282.9 ± 497.3	2,207.8 ± 694.1
Carbohydrate (g/kg)	4.0 ± 1.6	3.8 ± 1.2	3.7 ± 1.4
Fat (%)	29.5 ± 5.7	29.1 ± 3.1	29.4 ± 7.0
Protein (%)	20.7 ± 5.4	21.4 ± 3.8	22.4 ± 4.2
Calcium (mg)	868.3 ± 234.3	874.3 ± 426.7	811.4 ± 486.7
Iron (mg)	16.5 ± 4.2	15.3 ± 2.0	15.1 ± 4.5
Phosphorus (mg)	1,737.9 ± 458.9	1,748.8 ± 405.9	1,793.9 ± 771.2
Magnesium (mg)	345.0 ± 107.5	367.0 ± 108.0	340.0 ± 148.4
Selenium (µg)	37.8 ± 19.6	40.2 ± 18.3	48.9 ± 42.0
Zinc (mg)	16.1 ± 4.8	12.6 ± 3.9	62.9 ± 131.5
Vitamin A (µg)	229.6 ± 121.6	233.8 ± 122.6	216.0 ± 210.2
Vitamin B1 (mg)	1.2 ± 0.3	1.2 ± 0.4	1.3 ± 0.5
Vitamin B2 (mg)	1.5 ± 0.4	1.5 ± 0.6	1.5 ± 0.8
Vitamin B3 (mg)	28.9 ± 16.4	31.9 ± 11.9	33.2 ± 21.0
Vitamin B6 (mg)	1.0 ± 0.5	1.3 ± 0.6	1.2 ± 0.8
Vitamin B9 (µg)	183.1 ± 107.6	164.3 ± 61.8	150.6 ± 89.1
Vitamin B12 (µg)	1.1 ± 1.2	0.7 ± 0.9	1.1 ± 1.3
Vitamin C (mg)	133.0 ± 100.3	451.8 ± 743.4	105.7 ± 62.4
Vitamin D (µg)	0.8 ± 1.7	0.8 ± 2.0	0.9 ± 3.3
Vitamin E (mg)	0.6 ± 0.3	0.6 ± 0.4	0.6 ± 0.5

Data are presented as mean ± SD. There was no statistical difference (*P* > 0.05) for any of the dietary variable analyzed

*YMD-CHO* yerba mate drink and carbohydrate meal*, YMD-F* yerba mate drink and fasted state, *Control-CHO* control (water) and carbohydrate meal

### Blood Sampling

The venous blood was collected by venous puncture using a vacuum system (Vacutainer-BD, São Paulo, SP, Brazil) using lithium heparin tubes and tubes without anticoagulants or additives to obtain serum. The aliquots were obtained by centrifugation (1000 × g, 15 min; at 4 °C) to separate serum and plasma and promptly stored in microtubes (Eppendorf, Hamburg, Germany) at −80 °C for laboratory measurements. For blood lactate ([La^–^]) and glucose concentrations determination, 25 μL of capillary blood samples was collected from the earlobe using a heparinized capillary microtube. The samples were immediately analyzed with an electrochemical analyzer (YSI 2700, Yellow Springs, OH, USA).

### Gas Exchange Assessment

Respiratory exchanges (oxygen uptake [*V̇*O_2_], carbon dioxide production [*V̇*CO_2_], and ventilation [*V̇*E]) were measured breath-by-breath throughout a face mask during the exercise tests using gas analyzers (Quark CPET, COSMED, Rome, Italy; and K5, COSMED, Rome, Italy). Heart rate (HR) was recorded utilizing a chest strap (Garmin, Olathe, Kansas, USA) connected to the gas analyzer system. Two gas analyzers were used because of technical problems in one piece during data collection; however, it is noteworthy that all subjects completed all their respective tests using the same piece (Quark CPET, *n* = 3 and K5, *n* = 5). Both devices utilized are from the same manufacturer and have similar technology, and they showed to be valid compared to the Douglas Bag system [26, 27]. Before each test, the gas analyzers and flow meters were calibrated following the manufacturer’s instructions. Both analyzers were calibrated using the room air and an alpha standard gas of known concentration (16% oxygen and 5% carbon dioxide). The turbine flow meters were calibrated with a 3L calibration syringe. In addition, for K5 only, a delay calibration (i.e., the time required by the gas to reach the gas analyzer) was made.

### Metabolic Measurements and Gross Efficiency

During the cycling test on CLT and TT periods, the *V̇*O_2_ and *V̇*CO_2_ values were used to calculate the respiratory exchange ratio (RER) (*V̇*O_2_ ÷ *V̇*CO_2_), and fat and CHO oxidation rates according to the following equations [28]:

$${\text{Fat}}\;{\text{oxidation}}({\text{FAT}}_{{{\text{OX}}}} ) = (1.695 \times V{\text{O}}_{2} ) - (1.701 \times V{\text{CO}}_{2} )$$

$${\text{CHO}}\;{\text{oxidation}}({\text{CHO}}_{{{\text{OX}}}} ) = (4.21 \times V{\text{CO}}_{2} ) - (2.962 \times V{\text{O}}_{2} )$$

where *V̇*o_2_ and *V̇*co_2_ are in liters per minute (L/min) and oxidation rates are in grams per minute (g/min). Gross efficiency was calculated using the average values recorded during the entire 40-min CTL and 20-min TT (for both power output and gas exchange), using the method described by Moseley and Jeukendrup [29]:

$${\text{Energy}}\; {\text{expenditure}} \;\left( {J \cdot s^{ - 1} } \right) = \left[ {\left( {3.869 \times \dot{V}{\text{O}}_{2} } \right) + \left( {1.195 \times \dot{V}{\text{CO}}_{2} } \right)} \right] \times \left( {\frac{4.186}{{60}}} \right) \times 1000 \to$$

$$\mathrm{Gross}\, \mathrm{efficiency} \left(\%\right)= \frac{\mathrm{Power} \,\mathrm{output} (W)}{\mathrm{Energy} \,\mathrm{expenditure }\left(J \cdot {s}^{-1}\right)}\times 100$$

### Oxidative Stress Biomarkers

Plasma total phenolic concentration was determined spectrophotometrically according to the Folin–Ciocalteu method [24] and was expressed in μg of gallic acid per mL. The total antioxidant capacity (TAC) in the serum was evaluated according to the method described by Erel [30]. The results were expressed in mmol of the equivalent of Trolox per L. Serum total oxidant status (TOS) was measured using the previously published protocol proposed by Erel [31]. The results were expressed in μmol of hydrogen peroxide (H_2_O_2_) equivalent per L. The proportion of TOS by TAC was measured as the oxidative stress index (OSI) [32]. For the calculations, the value of OSI was obtained according to the following equation:

$$\mathrm{OSI }(\mathrm{arbitrary unit}) = \frac{TOS (\mathrm{\mu mol }\,{H}_{2}{O}_{2}\mathrm{ equiv}./\mathrm{L}) }\,{TAC (\mathrm{mmol \,Trolox \,equiv}./\mathrm{L})}$$

### Incremental Exercise Test

An incremental exercise test was performed on a cycle ergometer Lode Excalibur Sport (Lode BC, Groningen, The Netherlands). The test started at 100 W for 3 min and had increments of 30 W every 3 min until voluntary exhaustion. During the test, gas exchange data were continuously measured breath-by-breath. At the end of each 3-min stage (last 30 s), capillary blood samples were collected from the earlobe for [La^–^] determination. The *V̇*O_2_ data were plotted as a function of the time in averages of 30 s, and the highest value was considered the *V̇*O_2peak_. The MAP was determined as the power output (PO) of the last stage completed during the test [33]. From [La^–^] data were determined the first (LT_1_) and second (LT_2_) lactate thresholds. Briefly, LT_1_ was determined as the lowest equivalent between [La^–^] and PO (i.e., [La^–^] ÷ PO), and LT_2_ was determined as LT_1_ plus 1.5 mmol/L [34]. The PO at LT_1_ and LT_2_ was used in exercise performance protocol settings. All exercise sessions, including those of the experimental exercise protocol, were conducted employing the same cycle ergometer and under standardized laboratory conditions (~ 21 °C and relative humidity ~ 55%) and using a fan in front of the subjects with a standardized wind speed among the trials.

### Performance Test

The cycling exercise protocol had a total duration of 65 min. It started with a 40-min CLT at 95% of LT_1_ PO (i.e., 159 ± 23 W). Subsequently, without interruption, the subjects performed a 20-min TT test, followed by 2 min of rest, and finally, the subjects were instructed to perform four 10-s all-out sprints, each separated by 50-s recovery at 20 W (i.e., 4 × 10-s all-out: 50-s 20 W). For 40-min CLT, the subjects were instructed to select a preferred cadence in the first trial, to maintain it constant during the entire 40-min (± 5 RPM), and then to adopt a similar cadence among the subsequent trials. For 20-min TT and *all-out* sprints, subjects were instructed to produce the highest PO possible.

During the 20-min TT, the linear mode of the cycle ergometer was set. Thus, subjects completed in 20 min an amount of mechanical work (*Ẇ*) according to the following equation:

$$\dot{W} = L \times (RPM)^{2}$$

where *RPM* is the pedaling revolutions per minute and *L* is a linear factor. The linear factor was determined as:

$$L = \frac{{(RPM)}^{2} }{AP}$$

where *RPM* is the subjects’ preferred pedaling cadence and *AP* is the approximate expected PO for the duration of TT.

The AP was estimated as 110% of LT_2_ PO, based on a previous study performed in a similar cohort of cyclists [35]. The four 10-s *all-out* sprints were performed using the “Wingate” mode of the ergometer with a resistance factor of 0.075 × subject's body mass (kg). During 40-min CLT and 20-min TT, gas exchange and HR were continuously recorded. Capillary blood samples were obtained at rest, every 10 min during CLT, and every 5 min during TT. The rating of perceived exertion (RPE) by 15 points Borg scale [36] was administered every 8 min and 5 min during CLT and TT, respectively.

The subjects could drink water ad libitum during the tests. Only feedback provided during the tests was the real time in each part of the exercise protocol (i.e., CTL, TT, and sprints). However, other data (i.e., PO, *V̇*O_2_, HR, and cadence) were withheld to avoid possible influences on pacing strategy. Further, no verbal encouragement was provided [37]. Also, the subjects were not given access to their performance results until the research had concluded.

### Sample Size and Statistical Analysis

We conducted a sample size calculation in G*Power (Version 3.1.9.4, Germany) based on the intra-class correlation of 20-min TT of 0.96 [38], alpha of 5%, the statistical power of 80%, and a small F effect size (partial eta squared: 0.01); this yielded a sample size of 15 subjects.

The Shapiro–Wilk test was applied to check the normality of model residuals. Whenever possible, data that were not normally distributed were log-transformed. For normally distributed data, a two-way repeated-measures ANOVA model was used. The model fixed effects were the conditions (YMD-F, YMD-CHO, and Control-CHO), time points (every 4 and 2 min during CLT and TT, respectively, for *V̇*O_2_, *V̇*CO_2_, RER, FAT_ox_, CHO_ox_, HR, and PO; for [La^–^], glucose, and RPE the time points for each was described above), and the interaction intervention × time points. The random effects were the identity of each subject (because sample sizes in the conditions were not equal). Trials order and gross efficiency effects were analyzed using a mixed-model repeated-measures one-way ANOVA, models fixed effects were the order of the trials and conditions, respectively, and the random effects were the identity of each subject. When a fixed effect was significant, Tukey’s post hoc test was applied. For non-normally distributed data, the Friedman test with Tukey’s post hoc test was used for time analysis, and Wilcoxon’s signed-rank test was used to compare conditions at specific time points. For the oxidative stress and dietary intake variables, the Student’s paired *t* test or Wilcoxon’s signed-rank test (choice dependent on the normality test) was used to testing differences between conditions. Relationships between TT average PO and oxidative stress variables were examined with the Pearson correlation coefficient. Data were analyzed using R v3.6.3 software (R Core Team, Vienna, Austria). Statistical significance was set at *P* < 0.05. For normally distributed data, Cohen’s *d* effect size (ES) was estimated with an online available tool (sportsci.org/resource/stats/xcrossover.xls) [39] (https://www.biorxiv.org/content/10.1101/073999v2.supplementary-material). All data are expressed as mean ± standard deviations (SD), except for the graphics of oxidative stress variables (median and interquartile interval) and ES (mean ± 95% confidence interval).

## Results

### Subjects

Thirty-two male subjects were assessed for eligibility, and 14 subjects were excluded because they did not meet the inclusion criteria (*n* = 9) or declined to participate (*n* = 5). The randomized sample comprised 18 subjects, who were allocated in a crossover design to the three dietary trials. Among the 18 subjects, ten subjects had their tests interrupted due to COVID-19 lockdown after randomization. The final sample comprised eight trained cyclists (age 38 ± 3 years; height 1.75 ± 0.08 m; body mass 77.6 ± 8.0 kg; fat-free mass 60.9 ± 7.4 kg; fat mass 12.4 ± 4.6 kg; *V̇*O_2peak_ 56.5 ± 6.3 mL/kg/min; maximum aerobic power [MAP] 300 ± 43 Watts [W]). One participant dropped out of the study during the third period of the crossover trial when he would have received the YMD-F trial. Therefore, analyses were performed with *n* = 7 for the YMD-F trial and *n* = 8 for the YMD-CHO and Control-CHO trials.

### Performance and Perceptual Measurements

**Fig. 2:**
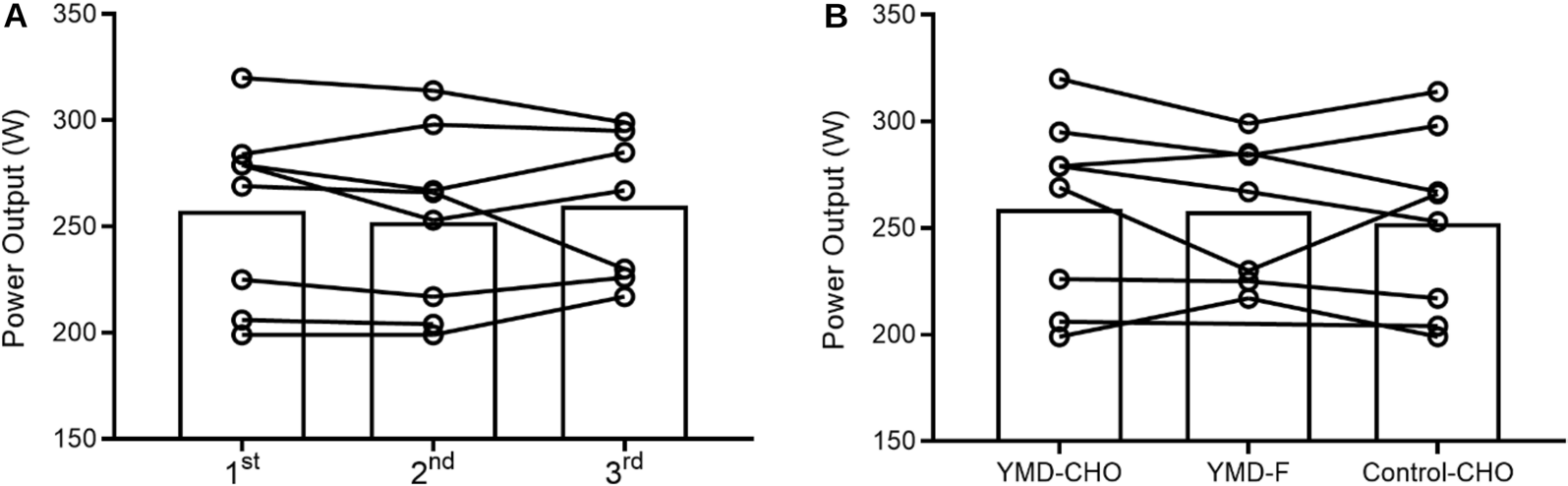
Individual data of average PO during TT for the trials order (**A**) and dietary conditions (**B**) effects. Open circles and lines are the data for each subject among the trials, bars are each trial/condition mean. *PO* power output, *TT* 20-min time trial, *YMD-CHO* yerba mate drink and carbohydrate meal, *YMD-F* yerba mate drink and fasted state, *Control-CHO* control (water) and carbohydrate meal

**Fig. 3:**
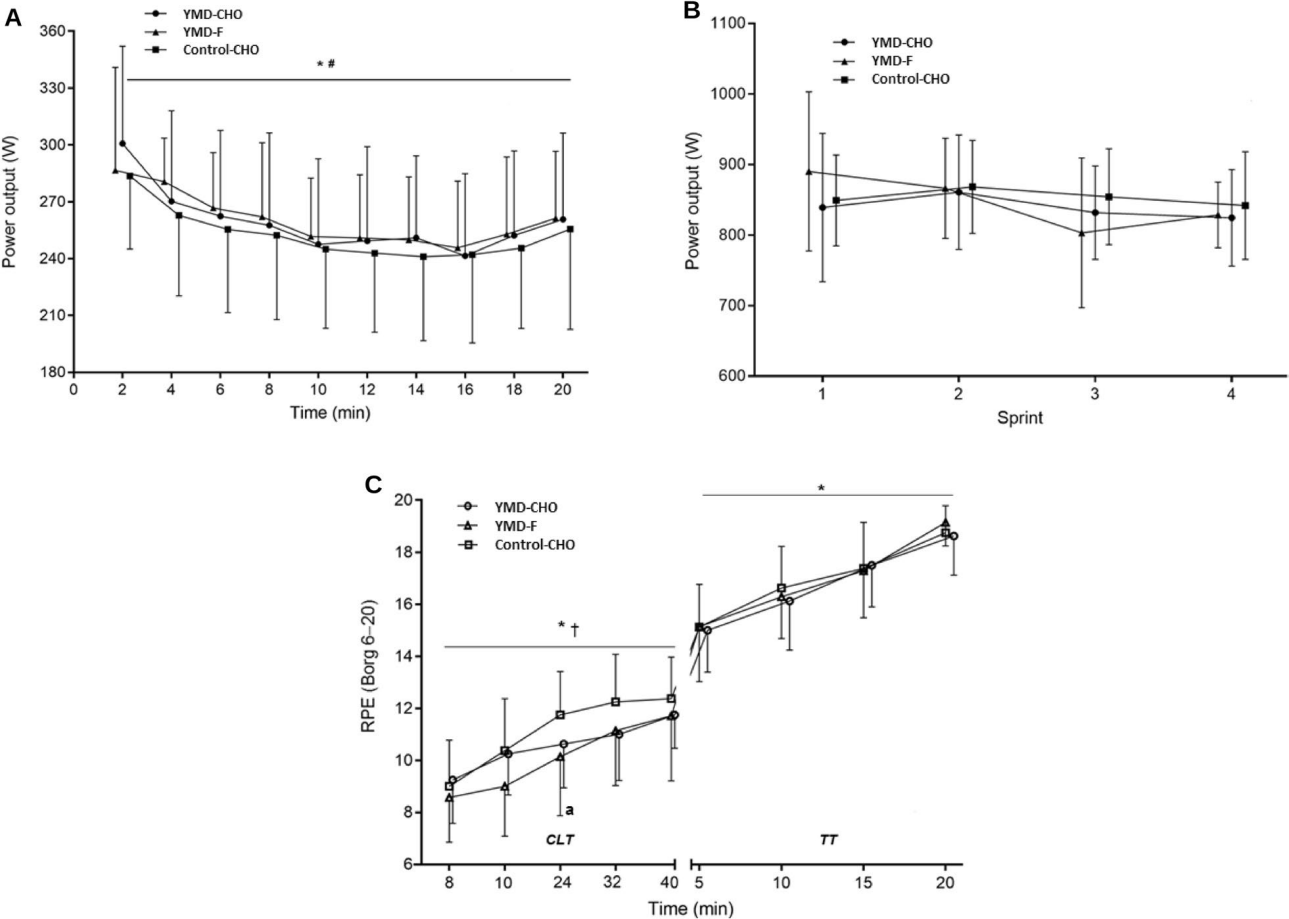
PO at each time point during TT (**A**) and during repeated sprint test (**B**). RPE during CLT followed by the TT (**C**). In panel A, “2 min” refers to the average of data between the minutes 0 and 2, “4 min” refers to minutes 0 and 4, and so on. YMD-CHO yerba mate drink and pre-exercise carbohydrate meal (*n* = 8), YMD-F yerba mate drink and fasted state (*n* = 7), Control-CHO control (water) and pre-exercise carbohydrate meal (*n* = 8). Data are means ± SD. *Significant time effect (*P* < 0.05). Significant condition effects (*P* < 0.05): #YMD-CHO > Control-CHO and †YMD-F > Control-CHO. ^a^Significatively different between YMD-F vs. Control-CHO (*P* < 0.05) at the specific time point. *CLT* 40-min constant load test, *PO* power output, *RPE* rating of perceived exertion, *TT* 20-min time trial

Descriptive data for performance and perceptual variables in CLT and TT are presented in Additional file 1. Figure 2A presents the order of the trials. There was no significant effect of trial order (*P* = 0.577), highlighting no familiarization effect among conditions. Individual average PO during TT among conditions is shown in Fig. 2B. There were time (*P* < 0.001) and condition (*P* = 0.018) effects for PO during 20-min TT. PO decreased over time, regardless of trial. The average PO was significantly higher (+ 3 ± 4%) in YMD-CHO than in Control-CHO, with a small ES (259 ± 44 W *vs*. 252 ± 43 W; *P* = 0.022; *d*: 0.20 ± 0.17). The average PO tended to be higher (*P* = 0.077; *d*: 0.16 ± 0.18) in YMD-CHO than in YMD-F (258 ± 33 W) (Fig. 3A). There were no differences between trials in average PO in the sprint test (843 ± 66 W, 847 ± 77 W, and 855 ± 42 W, for YMD-CHO, YMD-F, and Control-CHO, respectively; Fig. 3B), and in each of the four sprints (Additional file 1). During CLT, there were time (*P* < 0.001) and condition (*P* = 0.003) significant effects for RPE. RPE increased over time, regardless of trial. There was a small but significant decrease in RPE in YMD-F compared with Control-CHO (10 ± 2 *vs*. 11 ± 2; *P* = 0.002; *d*: 0.58 ± 0.36). During TT, RPE showed a time effect (*P* < 0.001), increasing over time in the three trials (Fig. 3C). There were no differences in RPE between trials after each of the 4 sprints (Additional file 1).

### Physiological Variables and Gross Efficiency

Descriptive data for physiological variables in CLT and TT are presented in Additional file 1. Average values for these variables during CLT and TT are outlined in Table 2.

**Table 2:** Averages for metabolic variables during CLT and TT in the YMD-CHO, YMD-F, and control-CHO trials

	CLT	TT
*FAT*_*ox*_* (g/min)*		
YMD-CHO	0.76 ± 0.18*^,#,^	0.70 ± 0.51*^,#^
YMD-F	0.71 ± 0.17*	0.36 ± 0.32
Control-CHO	0.64 ± 0.13	0.39 ± 0.29
*CHO*_*ox*_* (g/min)*		
YMD-CHO	1.06 ± 0.34	2.71 ± 0.75^#^
YMD-F	1.07 ± 0.32	3.28 ± 0.76*
Control-CHO	1.16 ± 0.19	2.91 ± 0.73
*V̇O*_2_ *(L/min)*		
YMD-CHO	2.39 ± 0.32*^,#^	3.56 ± 0.71*^,#^
YMD-F	2.30 ± 0.17	3.34 ± 0.47*
Control-CHO	2.22 ± 0.25	3.10 ± 0.39
*V̇CO*_2_ *(L/min)*		
YMD-CHO	1.93 ± 0.24	3.15 ± 0.45*
YMD-F	1.87 ± 0.12	3.13 ± 0.41*
Control-CHO	1.84 ± 0.19	2.87 ± 0.35
*GE (%)*		
YMD-CHO	19.8 ± 2.7	21.5 ± 4.2
YMD-F	20.9 ± 2.6	22.3 ± 2.5
Control-CHO	21.2 ± 2.3	23.4 ± 2.8
*RER*		
YMD-CHO	0.81 ± 0.02*	0.89 ± 0.08*^,#^
YMD-F	0.81 ± 0.03*	0.94 ± 0.06
Control-CHO	0.83 ± 0.02	0.93 ± 0.06
*HR (bpm)*		
YMD-CHO	123 ± 10	161 ± 14*
YMD-F	119 ± 12	160 ± 15*
Control-CHO	122 ± 15	158 ± 17

Data are presented as mean ± SD. *P < 0.05, compared with control-CHO; ^#^*P* < 0.05, compared with YMD-F; Significant condition effect (*P* < 0.05). *CLT* constant load test; *TT* time trial; FAT_ox_ fat oxidation; CHO_ox_ carbohydrate oxidation; *V̇*O_2_ oxygen uptake; *V̇*CO_2_ carbon dioxide production; *GE* gross efficiency; *RER* respiratory exchange ratio; *YMD-CHO* yerba mate drink and carbohydrate meal*, YMD-F* yerba mate drink and fasted state, *Control-CHO* control (water) and carbohydrate meal

#### CLT

**Fig. 4:**
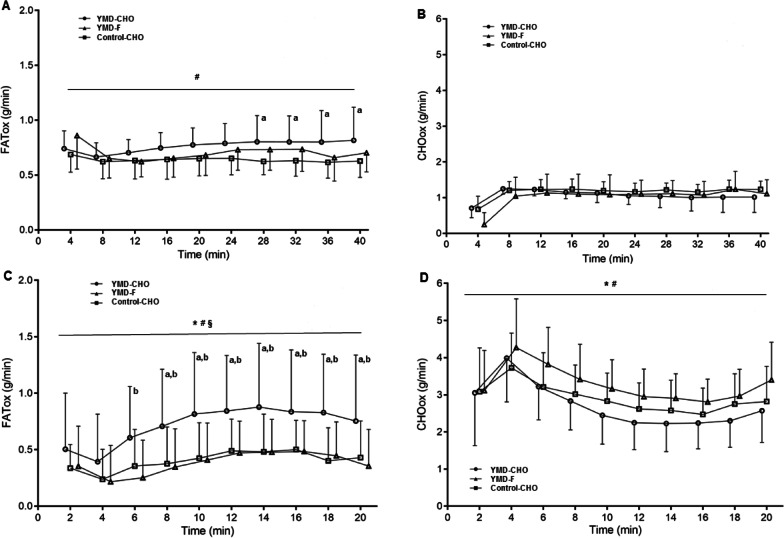
FAT_ox_ and CHO_ox_ during CLT (**A** and **B**), and during a subsequent TT (**C** and **D**). For CTL, “4 min” refers to the average of data between the minutes 0 and 4; for TT, “2 min” refers to minutes 0 and 2, and so on. Data are means ± SD. YMD-CHO yerba mate drink and pre-exercise carbohydrate meal (*n* = 8), YMD-F yerba mate drink and fasted state (*n* = 7), Control-CHO control (water) and pre-exercise carbohydrate meal (n = 8). *Significant time effect (*P* < 0.05). Significant condition effects (*P* < 0.05. ^#^*YMD-CHO* > *Control-CHO* and ^§^*YMD-CHO* > *YMD-F*. Significantly different between ^a^*YMD-CHO vs. Control-CHO* and ^b^*YMD-CHO vs. YMD-F* at the specific time point (*P* < 0.05). *FAT*_*ox*_ fat oxidation, *CHO*_*ox*_ carbohydrate oxidation, *CLT* 40-min constant load test, *TT* 20-min time trial

FAT_ox_ showed a condition effect (*P* < 0.001). FAT_ox_ in YMD-CHO was higher by ~ 13% than in YMD-F (P = 0.041; *d*: 0.50 ± 0.48), and by ~ 27% than in Control-CHO (*P* < 0.001; *d*: 0.95 ± 0.47). Also, FAT_ox_ was ~ 16% higher in YMD-F than in Control-CHO (*P* = 0.023; *d*: 0.64 ± 0.55) (Fig. 4A). During CLT there was a significant effect on CHO_ox_ in YMD-CHO and YMD-F (Friedman test: *P* = 0.002 and *P* = 0.014, respectively), with no differences between the three trials (Fig. 4B). *V̇*O_2_ showed time (*P* < 0.001) and condition (*P* < 0.001) effects and was higher in YMD-CHO than in both YMD-F and Control-CHO (*P* = 0.001; *d*: 0.49 ± 0.29 and *P* < 0.001; *d*: 0.70 ± 0.22, respectively). During CLT there was a significant effect on *V̇*CO_2_ in YMD-CHO, YMD-F, and Control-CHO (Friedman test: *P* = 0.017, *P* = 0.014, and *P* = 0.001, respectively), with no difference between trials. RER showed time (*P* < 0.001) and condition (*P* < 0.001) effects and was lower in both YMD-CHO and YMD-F than in Control-CHO (*P* < 0.001; *d*: 0.77 ± 0.41 and *P* = 0.011; *d*: 0.72 ± 0.55, respectively). Gross efficiency (Table 2) was not different among trials (*P* = 0.210). HR increased significantly in YMD-CHO, YMD-F, and Control-CHO (Friedman test: *P* < 0.001 for all tests), with no differences between trials.

#### TT

FAT_ox_ showed time (*P* < 0.001) and condition (*P* < 0.001) effects. In YMD-CHO, FAT_ox_ was higher by ~ 157% than in YMD-F (*P* < 0.001; *d*: 1.19 ± 0.26), and by ~ 151% than in Control-CHO (*P* < 0.001; *d*: 1.07 ± 0.26) (Fig. 4C). CHO_ox_ showed time (*P* < 0.001) and condition (*P* < 0.001) effects and was higher in YMD-F than in both YMD-CHO and Control-CHO (*P* = 0.001; *d*: 0.68 ± 0.40, and *P* = 0.037; *d*: 0.27 ± 0.25, respectively) (Fig. 4D). *V̇*O_2_ showed time (*P* = 0.001) and condition (*P* < 0.001) significant effects, it was higher in YMD-CHO than in both YMD-F and Control-CHO (*P* < 0.001; *d*: 0.74 ± 0.26, and *P* < 0.001; *d*: 1.18 ± 0.24, respectively). Also, *V̇*O_2_ was higher in YMD-F than in Control-CHO (*P* < 0.001; *d*: 0.48 ± 0.26). *V̇*CO_2_ showed time (*P* < 0.001) and condition (*P* < 0.001) effects and was higher in both YMD-CHO and YMD-F than in Control-CHO (*P* < 0.001; *d*: 0.79 ± 0.26 and *P* < 0.001; *d*: 0.24 ± 0.12, respectively). RER showed time (*P* < 0.001) and condition (*P* < 0.001) effects and was lower in YMD-CHO than in both YMD-F and Control-CHO (*P* < 0.001 *d*: 0.69 ± 0.27, and *P* < 0.001; *d*: 0.58 ± 0.24, respectively). Gross efficiency (Table 2) was not different among trials (*P* = 0.150). HR showed time (*P* < 0.001) and condition (*P* < 0.001) effects. HR was higher in both YMD-CHO and YMD-F than in Control-CHO (*P* = 0.002; *d*: 0.17 ± 0.11 and *P* < 0.001; *d*: 0.24 ± 0.13, respectively).

### Blood Glucose and Lactate

Details of blood metabolites are outlined in Table 3. Before exercise, blood glucose was lower in YMD-F than in both YMD-CHO and Control-CHO (*P* = 0.016; *δ*: 1.00 ± 0.55 and *P* = 0.016; *δ*: 0.96 ± 0.37, respectively). During CLT, glucose changed significantly, regardless of treatment (Friedman test: *P* < 0.001, P = 0.009, and *P* < 0.001, for YMD-CHO, YMD-F, and Control-CHO, respectively), but, on average, glucose was similar between trials. Compared with resting values, glucose was ~ 33–39% lower in YMD-CHO and Control-CHO from ~ 10 to 30 min, and ~ 16% higher in YMD-F at 40 min of CLT (Table 3). At 20 min, glucose values were higher in YMD-F than in both YMD-CHO and Control-CHO (0.73 ± 0.09 g/dL *vs.* 0.60 ± 0.10 g/dL; *P* = 0.047; *δ*: 0.68 ± 0.67 and 0.57 ± 0.10 g/dL; *P* = 0.016; *δ*: 0.68 ± 0.61, respectively). During TT, glucose increased significantly over time in YMD-CHO and YMD-F (Friedman test: *P* = 0.037 and *P* < 0.001, respectively), but with no differences between the three trials. Resting levels of [La^–^] were higher in YMD-F than in both YMD-CHO and Control-CHO (1.04 ± 0.30 mmol/L; *P* = 0.039; *δ*: 0.71 ± 0.61 and 1.42 ± 0.23 mmol/L; *P* = 0.047; *δ*: 0.75 ± 0.68, respectively). During CLT, [La^–^] did not change significantly, regardless of trial. During TT, [La^–^] showed time (*P* = 0.035) and condition (*P* = 0.002) effects. Average [La^–^] was higher in YMD-CHO and YMD-F than in Control-CHO (*P* = 0.004; *d*: 0.41 ± 0.28, and *P* = 0.011; *d*: 0.47 ± 0.36, respectively).

**Table 3:** Blood levels of glucose and lactate at pre-exercise and during CLT and TT in the YMD-CHO, YMD-F, and Control-CHO interventions

Variables	Rest	CLT					TT				
		10 min	20 min	30 min	40 min	Average	5 min	10 min	15 min	20 min	Average
*Glucose (g/dL)*											
YMD-CHO	0.97 ± 0.12^#^	0.59 ± 0.09^a^	0.60 ± 0.10^a^,*^,#^	0.66 ± 0.12	0.74 ± 0.12	0.65 ± 0.10	0.74 ± 0.13	0.82 ± 0.23	0.84 ± 0.28	0.96 ± 0.38	0.84 ± 0.35
YMD-F	0.65 ± 0.11*	0.70 ± 0.07	0.73 ± 0.09*	0.72 ± 0.12	0.76 ± 0.09^a^	0.73 ± 0.09	0.75 ± 0.10	0.85 ± 0.11	0.90 ± 0.20	1.02 ± 0.24^c^	0.88 ± 0.15
Control-CHO	1.00 ± 0.22	0.62 ± 0.13^a^	0.57 ± 0.10^a^	0.67 ± 0.12^a^	0.81 ± 0.14^b^	0.67 ± 0.11	0.79 ± 0.16	0.85 ± 0.19	0.87 ± 0.23	0.85 ± 0.26	0.84 ± 0.20
YMD-CHO	1.62 ± 0.59^#^	1.41 ± 0.72	1.32 ± 0.53	1.28 ± 0.43	1.25 ± 0.36	1.31 ± 0.47	7.57 ± 3.22	9.00 ± 4.25	8.42 ± 3.65	8.94 ± 3.21	8.48 ± 3.44*
YMD-F	1.04 ± 0.30*	1.38 ± 0.58	1.32 ± 0.45	1.25 ± 0.33	1.46 ± 0.63	1.36 ± 0.43	7.44 ± 3.32	8.77 ± 3.37	8.61 ± 3.40	9.27 ± 3.33*	8.52 ± 3.29*
Control-CHO	1.42 ± 0.23	1.48 ± 0.76	1.19 ± 0.47	1.16 ± 0.30	1.35 ± 0.39	1.29 ± 0.46	6.46 ± 3.26	7.71 ± 3.85	7.23 ± 3.35	7.02 ± 3.38	7.10 ± 3.40

Data are presented as mean ± SD. ^a^*P* < 0.05, compared with resting values; ^b^*P* < 0.05, compared with the time point 20 min during CLT; ^c^*P* < 0.05, compared with the time point 5 min during TT; ^*^P < 0.05, compared with control-CHO; ^#^*P* < 0.05, compared with YMD-F

*CLT* constant load test, *TT* time trial, *YMD-CHO* yerba mate drink and carbohydrate meal*, YMD-F* yerba mate drink and fasted state, *Control-CHO* control (water) and carbohydrate meal, *[La*^*–*^*]* blood lactate concentration

### Oxidative Stress Biomarkers

**Fig. 5:**
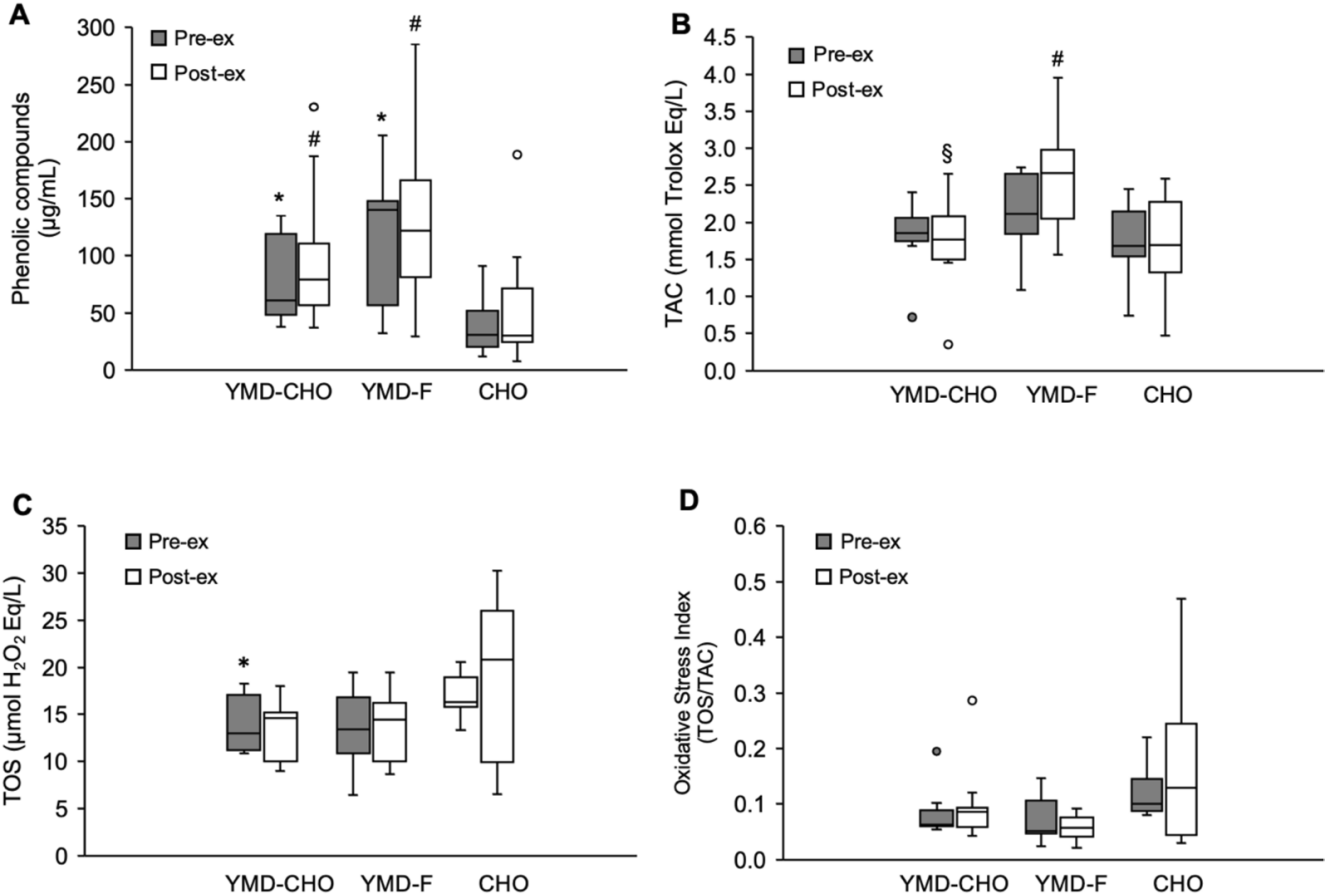
Phenolic compounds (**A**), TAC (**B**), TOS (**C**), and OSI (**D**) before (pre-ex) and after (post-ex) exercise protocol. YMD-CHO yerba mate drink and pre-exercise carbohydrate meal (n = 8), YMD-F yerba mate drink and fasted state (n = 7), Control-CHO control (water) and pre-exercise carbohydrate meal (n = 8). Data are median and interquartile interval. Outliers as . ^*^P < 0.05, compared with control-CHO at pre-ex. ^#^*P* < 0.05, compared with control-CHO at post-ex. ^§^*P* < 0.05, compared with YMD-F at post-ex. *TAC* serum total antioxidant capacity, *TOS* serum total oxidant status, *OSI* oxidative stress index

Details of oxidative stress variables are presented in Table 4. Before exercise, plasma phenolic compounds were higher compared with baseline (45.0 ± 25.4 μg/mL) in YMD-CHO and YMD-F (*P* = 0.002; *d* = 1.10 ± 0.42 and *P* = 0.004, *d* = 1.40 ± 0.60, respectively). Also, phenolics were higher in YMD-CHO and YMD-F than in Control-CHO (*P* = 0.004, *d* = 0.95 ± 0.41; *P* = 0.004, *d* = 1.43 ± 0.62, respectively) before exercise. After exercise, phenolics were higher in YMD-CHO and YMD-F than in Control-CHO (*P* = 0.004, *d* = 0.70 ± 0.52 and *P* = 0.050, *d* = 0.81 ± 0.81, respectively) (Fig. 5A). The serum levels of TAC, TOS, and OSI before exercise were not significantly different from baseline (1.78 ± 0.46 mmol Trolox Eq/L, 16.53 ± 4.95 μmol H_2_O_2_ Eq/L, and 0.10 ± 0.03, respectively), irrespective of trial. TAC values were not significantly different between trials before exercise and were higher in YMD-F than in YMD-CHO and Control-CHO after exercise (*P* = 0.030, *d* = 1.53 ± 1.03; *P* = 0.023, *d* = 1.27 ± 0.79, respectively) (Fig. 5B). Before exercise, TOS values were lower in YMD-CHO than in Control-CHO (*P* = 0.039, *d* = 1.70 ± 1.25), and were ~ 27% lower (not significant) in YMD-F than in Control-CHO. TOS values were not different between trials after exercise (Fig. 5C). OSI values were not significantly different between trials before exercise but tended to be lower in YMD-F than in YMD-CHO and Control-CHO after exercise (*P* = 0.066; *d* = 0.59 ± 0.64, and *P* = 0.066; *d* = 0.75 ± 0.81, respectively) (Fig. 5D). In YMD-CHO, average PO during TT was significantly correlated with changes (as a percentage of pre-exercise) in phenolics (*r* = −0.88; *P* < 0.001), TAC (*r* = −0.87; *P* = 0.005), and OSI (*r* = 0.76; *P* = 0.021) after exercise. No significant correlations were found for the YMD-F and Control-CHO trials.

**Table 4:** Antioxidant variables in the circulation before (rest) and after exercise protocol in the YMD-CHO, YMD-F, and Control-CHO conditions

	Rest	After exercise
*Total phenolics (μg/mL)*^*1*^		
YMD-CHO	82.1 ± 39.0^*^	99.3 ± 66.0^#^
YMD-F	114.7 ± 63.6^*^	131.3 ± 87.1^#^
Control-CHO	39.7 ± 28.1	59.7 ± 64.0
*TAC (Trolox Eq/L)*^*2*^		
YMD-CHO	1.82 ± 0.48	1.75 ± 0.65^§^
YMD-F	2.11 ± 0.59	2.61 ± 0.81^#^
Control-CHO	1.75 ± 0.59	1.71 ± 0.77
*TOS (μmol H*_*2*_*O*_*2*_* Eq/L)*^*2*^		
YMD-CHO	13.80 ± 3.00^*^	13.44 ± 3.14
YMD-F	13.62 ± 4.55	13.58 ± 4.50
Control-CHO	17.09 ± 2.50	18.51 ± 6.85
*OSI (TOS/TAC)*^*2*^		
YMD-CHO	0.08 ± 0.04	0.10 ± 0.07
YMD-F	0.07 ± 0.04	0.06 ± 0.03
Control-CHO	0.12 ± 0.05	0.17 ± 0.08

Data are presented as mean ± SD. ^*^P < 0.05, compared with Control-CHO at rest; ^#^*P* < 0.05, compared with Control-CHO after exercise; ^§^*P* < 0.05, compared with YMD-F after exercise; *TAC*, total antioxidant capacity; *TOS*, total oxidant status; *OSI*, oxidative stress index; ^1^Plasma; ^2^Serum; *YMD-CHO* yerba mate drink and carbohydrate meal*, YMD-F* yerba mate drink and fasted state, *Control-CHO* control (water) and carbohydrate meal

## Discussion

We demonstrated that the consumption of YMD (5 g of leaves in 250 mL/day for seven days and 40 min before exercise) following pre-exercise CHO intake resulted in higher FAT_ox_ during a low-intensity 40-min CLT and a 20-min TT compared to YMD or CHO alone. We also found a significant performance improvement during TT compared with a pre-exercise CHO intake alone in moderately trained endurance cyclists. The findings that YM effects on fat utilization and performance during exercise can be influenced by a pre-CHO meal may be useful in planning sports nutrition strategies. In addition, in YMD-CHO, TT performance was correlated with changes in the concentration of blood antioxidant markers after exercise.

Contrary to our hypothesis, the effect of YMD on FAT_ox_ was not impaired despite the intake of a relatively large amount of CHO (~ 80 g) 1 h before exercise. Instead, FAT_ox_ unexpectedly enhanced with YMD-CHO, irrespective of the submaximal exercise test. FAT_ox_ during CLT increased by ~ 27% and ~ 16% in YMD-CHO and YMD-F, respectively, compared to Control-CHO. Hence, the YMD-CHO effect on FAT_ox_ may be comparable, at least in part, to a range of 23–28% increase at low-to-moderate-intensity domains at 30–80% *V̇*O_2max_ [12, 13]; at steady state individualized intensities corresponding to maximal fat oxidation [11]; and at time trial cycling exercise [12]. Moreover, the apparent synergism between YMD and CHO meal during CLT seems to have been enhanced (i.e., > 150% increase) when exercise intensity was higher, considering that FAT_ox_ was superior during ~ 75% of the 20-min TT in YMD-CHO. Our findings were unexpected since the consumption of 75 g of glucose 45 min before an incremental test reduced the maximal rate of FAT_ox_ at ~ 60% of *V̇*O_2max_ by 28% in moderately trained cyclists [15]. The reasons for such discrepancy are unclear, but it is feasible to assume that concurrent effects on fat metabolism [40] between insulin [14, 15] and specific constituents of YM [2, 6, 10–13] might be involved. In this sense, the content of xanthines, chlorogenic acid, and other caffeoyl derivatives in YM might be good candidates [12]. Alkhatib et al. [13] suggested that the main effects of YM on FAT_ox_ might be in part explained by central mechanisms and glycogen sparing of caffeine [41] as well as other bioactive compounds present in YM, such as chlorogenic acids. However, the underlying mechanisms of the influence of YMD-with-CHO on FAT_ox_ during exercise should be accurately investigated.

CHO_ox_ was independent of the trial during CLT, despite the differences in FAT_ox_. This result agreed with the report that CHO_ox_ was similar between YM and placebo during 30-min low-intensity cycling, albeit FAT_ox_ was higher in YM [11]. In contrast, lower CHO_ox_ has been found in YM *vs*. placebo at intensities < 70% *V̇*O_2max_ during an incremental protocol in physically active subjects [13] and well-trained athletes [12]. In male endurance cyclists/triathletes, for instance, CHO_ox_ was higher in the placebo than in YM at 30%, 40%, and 50% *V̇*O_2max_ in a step test [12]. During low-to-moderate-intensity exercises, fat is the primary energy substrate but, as exercise intensity increases, FAT_ox_ gradually decreases, whereas CHO_ox_ rises. At heavy exercise intensities (≥ 75% *V̇*O_2max_), CHO is the predominant energy source, reaching saturation levels (i.e., RER = 1) at maximal intensities [42–44]. In the study mentioned above [11], CHO_ox_ during a low-intensity exercise initially increased and then decreased over time in YM and placebo, and this response paralleled FAT_ox_. Herein, after increasing initially, CHO_ox_ during CLT reached a plateau in the three trials, probably due to the similar response in FAT_ox_. One explanation for the differences between those studies [11–13] and ours might be the exercise protocol and the treatment trials. Interestingly, however, the pattern of inverse responses of CHO_ox_ and FAT_ox_ observed during low and moderate exercises in the fasted state [42] was quite clear during TT in YMD-CHO. In addition, despite CHO_ox_ decreasing during TT, regardless of treatment, average CHO_ox_ was higher in YMD-F than in the CHO trials. Thus, this is an evidence that YMD intake after an overnight fast may influence CHO_ox_ during a high-intensity exercise, and this effect seems to be attenuated by a previous CHO meal. It is possible, therefore, that increasing CHO availability through a pre-meal could help to spare glycogen stores [13] and may work well synergistically with YM in exercise intensities in the heavy domain. Furthermore, it is noteworthy that the higher lactate during the ~ 20-min TT in the YMD trials than in Control-CHO agreed with the report of higher plasma lactate in YM *vs*. placebo after 2/3 of a 30-min cycling TT [12]. However, lactate was similar between the YMD trials, suggesting that the higher CHO_ox_ in YMD-F *vs*. CHO trials involved an increment in mitochondrial metabolism. Taken together, these results suggest that changes in CHO metabolism elicited by YMD during high-intensity submaximal exercise may have implicated distinct patterns of the contribution of energy systems (i.e., anaerobic *vs*. aerobic metabolism [45]), according to feeding status (i.e., overnight fast *vs*. absorptive state). Finally, it is worth mentioning that during CLT glucose was better sustained in YMD-F than in Control-CHO, despite the lower resting levels in YMD-F. In addition, the higher glucose at the end of the 20-min TT compared with at rest in YMD-F agreed with the report of Areta et al. [12] that glucose raised above resting values after 2/3 of a 30-min TT in the YM condition. Hence, these results could suggest that pre-exercise YM intake may be a good dietary strategy to help to maintain blood glucose during exercise performed in the fasted state [18–20].

The effect of YM on TT performance was first demonstrated by Areta et al. [12]. The authors reported a small but significant improvement in PO (+ 2.3% ± 2.6%) in a ~ 30-min TT in YM than in placebo. Therefore, our finding of a small but significantly higher PO (~ 3%) in a 20-min TT in YMD-CHO than in Control-CHO seemed to agree with their findings [12] and support our hypothesis that CHO intake enhanced the YMD effect on cycling performance. In addition, it is noteworthy that there was a trend for higher performance during TT in YMD-CHO *vs*. YMD-F. Lastly, in line with previous findings [12], exercise economy during CLT and TT was not affected by YMD-CHO, despite higher-average *V̇*O_2_. Maximal performance in 10-s sprints was not influenced by the YMD trials which could be in part related to the extremely high neural/metabolic strain typically associated with a repeated sprint exercise [46]. We emphasize that sprints were performed probably under fatigue conditions stemming from the previous high-intensity exercise protocol [47], a situation that resembles sprinting to the finish or to pass other cyclists in most endurance cycling races [48]. Additionally, our results seem to be in line with the report of no effects of YM on maximal performance measured by peak power, *V̇*O_2max_, and peak lactate during an incremental test [13]. Herein, it is important to recognize that, because of the lack of a placebo trial, the enhancements in both TT performance and perceptual (i.e., RPE) variables may be imputed to a placebo effect. The notion that belief per se can be a powerful modulator of exercise performance is supported by a neurobiological basis [49]. Hence, it is probably that our cyclists did “believe” that their performance would be improved by YMD. However, an interesting question could remain: if performance improvement with YMD-CHO was biased by a placebo effect, why performance was not also superior with YMD consumed in the fasted state compared with the pre-CHO meal? Could have been the placebo effect influenced by athletes’ feeding status? Nevertheless, the mechanisms by which YM improves high-intensity performance are unknown. Areta et al. [12] proposed that the ergogenic effects of YM could in part be explained by synergism between caffeine and chlorogenic acids in stimulating the central nervous system, through an increase in dopamine levels. Therefore, one could assume that a potential ergogenic effect of YMD-CHO on TT performance might involve, at least in part, a synergism between peripheral and central actions of CHO [50], and specific YM compounds, such as caffeine [12, 51].

As expected, acute YMD consumption favorably affected blood antioxidant defense [52, 53], as indicated by the improvements in phenolic and TOS levels at pre-exercise compared with baseline. Furthermore, although oxidative stress variables were unaffected by exercise (i.e., pre-/post-), irrespective of trial, phenolic levels after exercise were higher in the two YMD trials, indicating that pre-YMD intake ensured higher levels of blood dietary antioxidants during exercise. Interestingly, changes in blood levels of phenolic and TAC induced by exercise were strongly and inversely associated with average PO during TT only in YMD-CHO. Although these findings do not represent cause–effect relationships, they might suggest that performance involved substantial recruitment of blood dietary antioxidants, mainly when athletes’ YMD intake was preceded by a pre-exercise CHO meal. In addition, it is noteworthy that, after exercise, TAC values were lower in YMD-CHO and Control-CHO *vs*. YMD-F, with large effect sizes. The reason for this finding was unclear. However, considering that there was no difference in phenolic levels between the YMD trials after exercise, we speculate that pre-exercise CHO meal may influence a possible recruitment of specific blood antioxidants during exercise performed after an overnight fast [54]. Nevertheless, associations between changes in blood oxidative stress markers and high-intensity performance must be further investigated.

Despite the interesting results, we recognize that the lack of a placebo-controlled condition is a major limitation in this study and may have influenced the significance of the findings, mainly regarding the performance and perceptual variables. YMD is a plant beverage that is challenging to imitate because of its sensory characteristics, and, thus, we could not find any suitable inert liquid substance that could act as a placebo. The small sample size is also a relevant limitation that restrained strong inferences about our results [55]. However, crossover studies with dietary clinical trials are generally composed of few participants, considering the commitment regarding lifestyle control variables during the study and the treatment intake. An additional limitation regarding our data acquisition refers to the use of two different gas analyzers, which could produce some biases in data. Nevertheless, both pieces of equipment are from the same manufacturer, use the same technology, were demonstrated to be valid [26, 27], and each subject had his gas exchanges monitored by the same device in all trials. Our findings also have gender limitations, since we only studied the effects in male subjects. Lastly, our subjects’ low dietary intake of CHO (~ 4.0 g/kg/day) [19] also represents a potential confounder. Nevertheless, our findings partially aligned with those from a study in which athletes consumed adequate amounts of CHO on the previous day before exercise [12].

## Conclusion

Pre-exercise CHO intake did not negate the effect of YMD on FAT_ox_ and 20-min TT performance. Instead, the results suggest a synergism between the two dietary strategies. Additionally, the improvement in 20-min TT performance induced by YMD-CHO may involve redox mechanisms. Hence, YMD consumption may be a good phenolic-rich dietary strategy for endurance athletes, especially those following methods of periodized CHO intake, which include training with high- and low-CHO availability (e.g., training fasted) [50]. Further studies should corroborate our findings in larger samples and include the YMD and CHO intake during exercise. Cellular mechanisms underlying YMD effects on metabolism and performance should be investigated further.

## Supplementary Information

**Additional file 1:** Physiological, perceptual, and performance variables assessed at different time points during CLT, TT, and sprints in the YMD-CHO, YMD-F, and Control-CHO conditions.

## Data Availability

The datasets generated during and/or analyzed during the current study are available from the corresponding author on reasonable request.
