# Supplementary material for: The Impact of Pre-Exercise Carbohydrate Meal on the Effects of Yerba Mate Drink on Metabolism, Performance, and Antioxidant Status in Trained Male Cyclists
Source: Sports Med Open. 2022 Jul 16;8:93. doi: 10.1186/s40798-022-00482-3 (PMC9287718; doi:10.1186/s40798-022-00482-3)
Supplement: Supplementary file 1 — Additional file 1: Physiological, perceptual, and performance variables assessed at different time points during CLT, TT, and sprints in the YMD-CHO, YMD-F, and Control-CHO conditions. [file 40798_2022_482_MOESM1_ESM.docx]

**The impact of pre-exercise carbohydrate meal on the effects of yerba mate drink on metabolism, performance, and antioxidant status in trained male cyclists**

*Sports Medicine Open*

Thaiana C. Krolikowski^1^, Fernando K. Borszcz^3^, Vilma Pereira Panza^1^, Laura M. Bevilacqua^2^, Sarah Nichele^2^, Edson L. da Silva^1,4^, Renata D. M. C. Amboni^5^, Luiz G. A. Guglielmo^1,3^, Stuart M. Phillips^6^, Ricardo D. de Lucas^3^, Brunna C. B. Boaventura^1,2*^

^1^ Graduate Program in Nutrition, Health Sciences Center, Federal University of Santa Catarina, Campus Trindade, Florianópolis, SC 88040-370, Brazil

^2^ Department of Nutrition, Health Sciences Center, Federal University of Santa Catarina, Campus Trindade, Florianópolis, SC 88040-370, Brazil

^3^ Physical Effort Laboratory, Sports Center, Federal University of Santa Catarina, Campus Trindade, Florianópolis, SC 88040-900, Brazil

^4^ Department of Clinical Analyses, Health Sciences Center, Federal University of Santa Catarina, Campus Trindade, Florianópolis, SC 88040-370, Brazil

^5^ Department of Food Science and Technology, Agricultural Sciences Center, Federal University of Santa Catarina, Campus Itacorubi, Florianópolis, SC 88034-001, Brazil

^6^ Department of Kinesiology, McMaster University, Hamilton, ON L8S 4K1, Canada

*Correspondence: brunnab@gmail.com/brunna.boaventura@ufsc.br

Department of Nutrition, Health Sciences Center, Federal University of Santa Catarina, Campus Trindade, Florianópolis, SC 88040-370, Brazil

**Table S1** Physiological and perceptual variables assessed at different time points during CLT in the YMD-CHO, YMD-F, and Control-CHO conditions.

| **Variables** | **CLT time points** | | | | | | | | | |
| --- | --- | --- | --- | --- | --- | --- | --- | --- | --- | --- |
|  | **4 min** | **8 min** | **12 min** | **16 min** | **20 min** | **24 min** | **28 min** | **32 min** | **36 min** | **40 min** |
| **FAT_ox_ (g/min)** | | | | | | | | | | |
| YMD-CHO | 0.74 ± 0.16 | 0.66 ± 0.13 | 0.70 ± 0.12 | 0.75 ± 0.14 | 0.77 ± 0.15 | 0.79 ± 0.18 | 0.80 ± 0.24* | 0.80 ± 0.24* | 0.80 ± 0.29* | 0.82 ± 0.30* |
| YMD-F | 0.86 ± 0.30 | 0.65 ± 0.18 | 0.62 ± 0.14 | 0.65 ± 0.17 | 0.68 ± 0.19 | 0.73 ± 0.19 | 0.73 ± 0.19 | 0.73 ± 0.20 | 0.66 ± 0.21 | 0.70 ± 0.17 |
| Control-CHO | 0.69 ± 0.16 | 0.62 ± 0.16 | 0.63 ± 0.17 | 0.64 ± 0.18 | 0.65 ± 0.16 | 0.65 ± 0.15 | 0.62 ± 0.12 | 0.63 ± 0.14 | 0.62 ± 0.15 | 0.63 ± 0.15 |
| **CHO_ox_ (g/min)** | | | | | | | | | | |
| YMD-CHO | 0.71 ± 0.27 | 1.25 ± 0.15^a^ | 1.23 ± 0.18^a^ | 1.15 ± 0.18^a^ | 1.13 ± 0.27^a^ | 1.05 ± 0.24 | 1.04 ± 0.32 | 1.00 ± 0.38 | 1.02 ± 0.43 | 1.01 ± 0.43 |
| YMD-F | 0.25 ± 0.34 | 1.04 ± 0.53 | 1.13 ± 0.53^a^ | 1.10 ± 0.56^a^ | 1.08 ± 0.56^a^ | 1.10 ± 0.39 | 1.11± 0.37 | 1.07 ± 0.39 | 1.23 ± 0.50^a^ | 1.11 ± 0.39 |
| Control-CHO | 0.74 ± 0.34 | 1.27 ± 0.17 | 1.29 ± 0.24 | 1.30 ± 0.24^a^ | 1.25 ± 0.22 | 1.16 ± 0.24 | 1.22 ± 0.19 | 1.16 ± 0.22 | 1.23 ± 0.25 | 1.23 ± 0.23 |
| ***V̇*O_2_ (L/min)** | | | | | | | | | | |
| YMD-CHO | 2.06 ± 0.40 | 2.34 ± 0.33^a^ | 2.41 ± 0.31^a^ | 2.43 ± 0.28^a^ | 2.47 ± 0.33^a^ | 2.44 ± 0.31^a^ | 2.45 ± 0.35^a,^* | 2.42 ± 0.34^a,^* | 2.43 ± 0.38^a^ | 2.46 ± 0.38^a^ |
| YMD-F | 1.89 ± 0.31 | 2.30 ± 0.17^a^ | 2.32 ± 0.17^a^ | 2.35 ± 0.17^a^ | 2.39 ± 0.15^a^ | 2.36 ± 0.16^a^ | 2.37 ± 0.21^a^ | 2.34 ± 0.22^a^ | 2.32 ± 0.26^a^ | 2.31 ± 0.24^a^ |
| Control-CHO | 1.93 ± 0.34 | 2.23 ± 0.31^a^ | 2.27± 0.26^a^ | 2.29 ± 0.27^a^ | 2.27 ± 0.24^a^ | 2.25 ± 0.23^a^ | 2.24 ± 0.22^a^ | 2.21 ± 0.29^a^ | 2.24 ± 0.21^a^ | 2.26 ± 0.23^a^ |
| ***V̇*CO_2_ (L/min)** | | | | | | | | | | |
| YMD-CHO | 1.62 ± 0.31 | 1.95 ± 0.26 | 1.99 ± 0.25^a^ | 1.99 ± 0.21 | 2.01 ± 0.26^a^ | 1.97 ± 0.23 | 1.97 ± 0.24 | 1.94 ± 0.25 | 1.95 ± 0.27 | 1.97 ± 0.25 |
| YMD-F | 1.38 ± 0.18 | 1.90 ± 0.09 | 1.94 ± 0.10 | 1.96 ± 0.11 | 1.98 ± 0.08^a^ | 1.92 ± 0.13 | 1.93 ± 0.17 | 1.90 ± 0.17 | 1.93 ± 0.24 | 1.89 ± 0.21 |
| Control-CHO | 1.52 ± 0.29 | 1.85 ± 0.24 | 1.89 ± 0.20^a^ | 1.91 ± 0.20^a^ | 1.88 ± 0.18^a^ | 1.86 ± 0.17 | 1.87 ± 0.17 | 1.83 ± 0.23 | 1.87 ± 0.16 | 1.88 ± 0.17 |
| **RER** | | | | | | | | | | |
| YMD-CHO | 0.78 ± 0.03^#^ | 0.83 ± 0.01^a^ | 0.82 ± 0.01 | 0.82 ± 0.02 | 0.81 ± 0.02 | 0.81 ± 0.03 | 0.81 ± 0.03 | 0.80 ± 0.04 | 0.81 ± 0.04 | 0.81 ± 0.05 |
| YMD-F | 0.73 ± 0.05* | 0.83 ± 0.04^a^ | 0.84 ± 0.03^a^ | 0.83 ± 0.04^a^ | 0.83 ± 0.04^a^ | 0.81 ± 0.04^a^ | 0.81 ± 0.04^a^ | 0.81 ± 0.04^a^ | 0.83 ± 0.05^a^ | 0.82 ± 0.04^a^ |
| Control-CHO | 0.78 ± 0.04 | 0.83 ± 0.03^a^ | 0.84 ± 0.04^a^ | 0.83 ± 0.04^a^ | 0.83 ± 0.03^a^ | 0.83 ± 0.03^a^ | 0.83 ± 0.02^a^ | 0.83 ± 0.03^a^ | 0.84 ± 0.03^a^ | 0.84 ± 0.03^a^ |
|  |  |  |  |  |  |  |  |  |  | (Continued) |

**Table S1** (Continued)

| **Variables** | **CLT time points** | | | | | | | | | |
| --- | --- | --- | --- | --- | --- | --- | --- | --- | --- | --- |
|  | **4 min** | **8 min** | **12 min** | **16 min** | **20 min** | **24 min** | **28 min** | **32 min** | **36 min** | **40 min** |
| **HR (bpm)** | | | | | | | | | | |
| YMD-CHO | 109 ± 16 | 122 ± 10 | 124 ± 10 | 124 ± 10 | 124 ± 10 | 124 ± 10 | 125 ± 10^a^ | 125 ± 10^a^ | 127 ± 10^a,b^ | 128 ± 11^a,b^ |
| YMD-F | 99 ± 5 | 113 ± 12 | 117 ± 14 | 117 ± 12 | 119 ± 14 | 121 ± 13 | 121 ± 13 | 122 ± 13^a^ | 124 ± 12^a,b^ | 124 ± 13^a,b^ |
| Control-CHO | 110 ± 13 | 121 ± 15 | 123 ± 14 | 124 ± 15^a^ | 123 ± 15 | 123 ± 14 | 124 ± 16^a^ | 123 ± 15 | 126 ± 16^a^ | 127 ± 16^a,b^ |
| **RPE (Borg 6−20)** |  |  |  |  |  |  |  |  |  |  |
| YMD-CHO | ⎯ | 9.3 ± 1.7 | ⎯ | 10.3 ± 1.6 | ⎯ | 10.6 ± 1.7 | ⎯ | 11.0 ± 1.8 | ⎯ | 11.8 ± 1.3^a^ |
| YMD-F | ⎯ | 8.6 ± 1.7 | ⎯ | 9.0 ± 1.9 | ⎯ | 10.1 ± 2.3* | ⎯ | 11.1 ± 2.1^a,c^ | ⎯ | 11.7 ± 2.5^a,c^ |
| Control-CHO | ⎯ | 9.0 ± 1.8 | ⎯ | 10.4 ± 2.0 | ⎯ | 11.8 ± 1.7^a^ | ⎯ | 12.3 ± 1.8^a,c^ | ⎯ | 12.4 ± 1.6^a,c^ |

*CLT* constant load test, *FAT_ox_* fat oxidation, *CHO_ox_* carbohydrate oxidation, *V̇O_2_* oxygen uptake, *V̇CO_2_* carbon dioxide production, *RER* respiratory exchange ratio, *HR* heart rate, *RPE* rating of perceived exertion, (⎯) not applicable, *YMD-CHO* yerba mate drink and carbohydrate meal*, YMD-F* yerba mate drink and fasted state, *Control-CHO* control (water) and carbohydrate meal. Data are presented as mean ± SD. ^a^*P* < 0.05, compared with the respective first time point determined during CLT; ^b^*P* < 0.05, compared with the time point 8 min during CL; ^c^*P* < 0.05, compared with the time point 16 min during CLT; ^*^*P* < 0.05, compared with control-CHO; ^#^*P* < 0.05, compared with YMD-F.

**Table S2** Physiological, perceptual, and performance variables assessed at different time points during TT and sprints in the YMD-CHO, YMD-F, and Control-CHO conditions.

| **Variables** | **TT time points** | | | | | | | | | | | | **Repeated sprint test** | | | |
| --- | --- | --- | --- | --- | --- | --- | --- | --- | --- | --- | --- | --- | --- | --- | --- | --- |
|  | **2 min** | **4 min** | **5 min** | **6 min** | **8 min** | **10 min** | **12 min** | **14 min** | **15 min** | **16 min** | **18 min** | **20 min** | **S1** | **S2** | **S3** | **S4** |
| **PO (W)** | | | | | | | | | | | | | | | | |
| YMD-CHO | 301 ± 51^#^ | 270 ± 48^a^ | ⎯ | 262 ± 45^a^ | 258 ± 49^a^ | 248 ± 45^a^ | 249 ± 50^a^ | 251 ± 43^a^ | ⎯ | 242 ± 43^a,b^ | 252 ± 45^a^ | 261 ± 46^a^ | 839 ± 105 | 861 ± 81 | 832 ± 66 | 825 ± 69 |
| YMD-F | 287 ± 54 | 281 ± 23 | ⎯ | 267 ± 29 | 262 ± 39 | 252 ± 31^a,b^ | 251 ± 33^a,b^ | 250 ± 44^a,b^ | ⎯ | 246 ± 35^a,b^ | 253 ± 41^a,b^ | 261 ± 35 | 890 ± 113 | 866 ± 71 | 803 ± 106 | 829 ± 47 |
| Control-CHO | 284 ± 39 | 263 ± 43 | ⎯ | 255 ± 44^a^ | 252 ± 45^a^ | 245 ± 42^a^ | 243 ± 42^a^ | 241 ± 33^a^ | ⎯ | 242 ± 47^a^ | 246 ± 42^a^ | 256 ± 53^a^ | 849 ± 65 | 868 ± 66 | 854 ± 68 | 842 ± 76 |
| **FAT_ox_ (g/min)** | | | | | | | | | | | | | | | | |
| YMD-CHO | 0.50 ± 0.50 | 0.39 ± 0.42^a^ | ⎯ | 0.61 ± 0.45^#^ | 0.71 ± 0.51*^,#^ | 0.82 ± 0.54^b,^*^,#^ | 0.84 ± 0.49^b,^*^,#^ | 0.88 ± 0.56^b,^*^,#^ | ⎯ | 0.84 ± 0.55^b,^*^,#^ | 0.83 ± 0.52^b,^*^,#^ | 0.75 ± 0.58*^,#^ | ⎯ | ⎯ | ⎯ | ⎯ |
| YMD-F | 0.35 ± 0.36 | 0.22 ± 0.32 | ⎯ | 0.25 ± 0.33 | 0.35 ± 0.34 | 0.41 ± 0.33 | 0.47 ± 0.28 | 0.48 ± 0.29 | ⎯ | 0.48 ± 0.27 | 0.45 ± 0.30 | 0.36 ± 0.32 | ⎯ | ⎯ | ⎯ | ⎯ |
| Control-CHO | 0.34 ± 0.21 | 0.24 ± 0.27 | ⎯ | 0.36 ± 0.32 | 0.38 ± 0.33 | 0.42 ± 0.31 | 0.49 ± 0.28 | 0.48 ± 0.33 | ⎯ | 0.50 ± 0.26 | 0.40 ± 0.29 | 0.43 ± 0.32 | ⎯ | ⎯ | ⎯ | ⎯ |
| **CHO_ox_ (g/min)** | | | | | | | | | | | | | | | | |
| YMD-CHO | 3.05 ± 1.42 | 3.99 ± 1.18 | ⎯ | 3.22 ± 0.90 | 2.83 ± 0.78^b^ | 2.45 ± 0.78^b^ | 2.25 ± 0.73^b^ | 2.23 ± 0.76^b^ | ⎯ | 2.24 ± 0.69^b^ | 2.30 ± 0.72^b^ | 2.57 ± 0.85^b^ | ⎯ | ⎯ | ⎯ | ⎯ |
| YMD-F | 3.11 ± 1.08 | 4.27 ± 1.31^a^ | ⎯ | 3.82 ± 1.00 | 3.41 ± 0.95 | 3.16 ± 0.79 | 2.95 ± 0.74^b^ | 2.91 ± 0.66^b^ | ⎯ | 2.81 ± 0.62^b^ | 2.97 ± 0.72^b^ | 3.40 ± 1.02 | ⎯ | ⎯ | ⎯ | ⎯ |
| Control-CHO | 3.08 ± 1.18 | 3.73 ± 0.93 | ⎯ | 3.21 ± 0.92 | 3.02 ± 0.79 | 2.83 ± 0.75 | 2.62 ± 0.70^b^ | 2.58 ± 0.82^b^ | ⎯ | 2.47 ± 0.71^b^ | 2.75 ± 0.82 | 2.82 ± 0.95 | ⎯ | ⎯ | ⎯ | ⎯ |
|  |  |  |  |  |  |  |  |  |  |  |  |  |  | (Continued) | | |

**Table S2** (continued)

| **Variables** | **TT time points** | | | | | | | | | | | | **Repeated sprint test** | | | | | |
| --- | --- | --- | --- | --- | --- | --- | --- | --- | --- | --- | --- | --- | --- | --- | --- | --- | --- | --- |
|  | **2 min** | **4 min** | **5 min** | **6 min** | **8 min** | **10 min** | **12 min** | **14 min** | **15 min** | **16 min** | **18 min** | **20 min** | **S1** | | | **S2** | **S3** | **S4** |
| ***V̇*O_2_ (L/min)** | | | | | | | | | | | | | | | | | | |
| YMD-CHO | 3.25 ± 0.46 | 3.70 ± 0.67* | ⎯ | 3.68 ± 0.70* | 3.67 ± 0.70* | 3.62 ± 0.72* | 3.52 ± 0.75* | 3.57 ± 0.80* | ⎯ | 3.50 ± 0.79* | 3.53 ± 0.82* | 3.54 ± 0.86* | | ⎯ | | ⎯ | ⎯ | ⎯ |
| YMD-F | 3.09 ± 0.31 | 3.47 ± 0.38 | ⎯ | 3.47 ± 0.44 | 3.43 ± 4.93 | 3.38 ± 0.52 | 3.34 ± 0.52 | 3.32 ± 0.53 | ⎯ | 3.25 ± 0.53 | 3.30 ± 0.58 | 3.32 ± 0.62 | | ⎯ | | ⎯ | ⎯ | ⎯ |
| Control-CHO | 2.97 ± 0.39 | 3.25 ± 0.33 | ⎯ | 3.21 ± 0.36 | 3.18± 0.39 | 3.15 ± 0.36 | 3.11 ± 0.40 | 3.06 ± 0.41 | ⎯ | 3.01 ± 0.47 | 3.04 ± 0.46 | 3.05 ± 0.48 | | ⎯ | | ⎯ | ⎯ | ⎯ |
| ***V̇*CO_2_ (L/min)** | | | | | | | | | | | | | | | | | | |
| YMD-CHO | 3.01 ± 0.42 | 3.55 ± 0.47^a,^* | ⎯ | 3.37 ± 0.48 | 3.26 ± 0.46 | 3.13 ± 0.49 | 3.01 ± 0.55^b^ | 3.04 ± 0.55^b^ | ⎯ | 2.99 ± 0.53^b^ | 3.03 ± 0.59^b^ | 3.11 ± 0.59 | | ⎯ | | ⎯ | ⎯ | ⎯ |
| YMD-F | 2.92 ± 0.33 | 3.45 ± 0.40^a^ | ⎯ | 3.35 ± 0.44 | 3.23 ± 0.49 | 3.13± 0.47 | 3.05 ± 0.48 | 3.02 ± 0.46 | ⎯ | 2.95 ± 0.46^b^ | 3.02 ± 0.51 | 3.14 ± 0.54 | | ⎯ | | ⎯ | ⎯ | ⎯ |
| Control-CHO | 2.82 ± 0.46 | 3.17 ± 0. 30 | ⎯ | 3.02 ± 0.35 | 2.95 ± 0.36 | 2.89 ± 0.35 | 2.81 ± 0.38 | 2.76 ± 0.39 | ⎯ | 2.71 ± 0.44^b^ | 2.79 ± 0.45 | 2.82 ± 0.44 | | ⎯ | | ⎯ | ⎯ | ⎯ |
| **RER** | | | | | | | | | | | | | | | | | | |
| YMD-CHO | 0.93 ± 0.12 | 0.97 ± 0.11 | ⎯ | 0.92 ± 0.09 | 0.90 ± 0.08 | 0.87 ± 0.07^b^ | 0.86 ± 0.07^b^ | 0.86 ± 0.08^b^ | ⎯ | 0.87 ± 0.07^b^ | 0.87 ± 0.07^b^ | 0.89 ± 0.09^b^ | | | ⎯ | ⎯ | ⎯ | ⎯ |
| YMD-F | 0.94 ± 0.09 | 1.00 ± 0.10 | ⎯ | 0.97 ± 0.07 | 0.94 ± 0.06 | 0.93 ± 0.05 | 0.91 ± 0.04^b^ | 0.91 ± 0.05^b^ | ⎯ | 0.91 ± 0.04^b^ | 0.92 ± 0.05^b^ | 0.95 ± 0.09 | | | ⎯ | ⎯ | ⎯ | ⎯ |
| Control-CHO | 0.95 ± 0.09 | 0.98 ± 0.08 | ⎯ | 0.95 ± 0.09 | 0.93 ± 0.06 | 0.92 ± 0.06 | 0.90 ± 0.05 | 0.91 ± 0.07 | ⎯ | 0.90 ± 0.05^b^ | 0.92 ± 0.06 | 0.93 ± 0.08 | | | ⎯ | ⎯ | ⎯ | ⎯ |
| **HR (bpm)** | | | | | | | | | | | | | | | | | | |
| YMD-CHO | 150 ± 17 | 158 ± 15 | ⎯ | 162 ± 14^a^ | 162 ± 15^a^ | 162 ± 14^a^ | 161 ± 15^a^ | 163 ± 14^a^ | ⎯ | 163 ± 14^a^ | 164 ± 15^a^ | 166 ± 14^a^ | | | ⎯ | ⎯ | ⎯ | ⎯ |
| YMD-F | 148 ± 19 | 159 ± 17^a^ | ⎯ | 160 ± 16^a^ | 161 ± 15^a^ | 161 ± 15^a^ | 161 ± 15^a^ | 163 ± 15^a^ | ⎯ | 161 ± 14^a^ | 164 ± 14^a^ | 166 ± 14^a^ | | | ⎯ | ⎯ | ⎯ | ⎯ |
| Control-CHO | 148 ± 20 | 157 ± 19 | ⎯ | 158 ± 19 | 159 ± 18^a^ | 159 ± 17^a^ | 159 ± 16^a^ | 159 ± 16^a^ | ⎯ | 159 ± 16^a^ | 161 ± 17^a^ | 162 ± 16^a^ | | | ⎯ | ⎯ | ⎯ | ⎯ |
|  |  |  |  |  |  |  |  |  |  |  |  |  | | |  | (Continued) | | |

**Table S2** (continued)

| **Variables** | **TT time points** | | | | | | | | | | | | **Repeated sprint test** | | | |
| --- | --- | --- | --- | --- | --- | --- | --- | --- | --- | --- | --- | --- | --- | --- | --- | --- |
|  | **2 min** | **4 min** | **5 min** | **6 min** | **8 min** | **10 min** | **12 min** | **14 min** | **15 min** | **16 min** | **18 min** | **20 min** | **S1** | **S2** | **S3** | **S4** |
| **RPE (Borg 6−20)** | | | | | | | | | | | | | | | | |
| YMD-CHO | ⎯ | ⎯ | 15.0 ± 1.6 | ⎯ | ⎯ | 16.1 ± 1.9 | ⎯ | ⎯ | 17.5 ± 1.6^a,c^ | ⎯ | ⎯ | 18.6 ± 1.5^a,c^ | ⎯ | ⎯ | ⎯ | ⎯ |
| YMD-F | ⎯ | ⎯ | 15.1 ± 2.1 | ⎯ | ⎯ | 16.3 ± 1.6 | ⎯ | ⎯ | 17.3 ± 1.8^a^ | ⎯ | ⎯ | 19.1 ± 0.9^a,c^ | ⎯ | ⎯ | ⎯ | ⎯ |
| Control-CHO | ⎯ | ⎯ | 15.1 ± 1.6 | ⎯ | ⎯ | 16.6 ± 1.6^a^ | ⎯ | ⎯ | 17.4 ± 1.8^a^ | ⎯ | ⎯ | 18.8 ± 1.0^a,c^ | ⎯ | ⎯ | ⎯ | ⎯ |

*Note:* The “2 min” refers to the average of data between the minutes 0 and 2, “4 min” to 2 and 4, and so on, except for RPE which was collected in the exact minute.

*TT* time-trial, *S* sprint, *PO* power output, *FAT_ox_* fat oxidation, *CHO_ox_* carbohydrate oxidation, *V̇O_2_* oxygen uptake, *V̇CO_2_* carbon dioxide production, *RER* respiratory exchange ratio, *HR* heart rate, *RPE* rating of perceived exertion, (⎯) not applicable, *YMD-CHO* yerba mate drink and carbohydrate meal*, YMD-F* yerba mate drink and fasted state, *Control-CHO* control (water) and carbohydrate meal. Data are presented as mean ± SD. ^a^*P* < 0.05, compared with the respective first time point determined during TT; ^b^*P* < 0.05, compared with the time point 4 min during TT; ^c^*P* < 0.05, compared with the time point 10 min during TT; ^*^*P* < 0.05, compared with Control-CHO; ^#^*P* < 0.05, compared with YMD-F.
